# Prevalence and Complications of Pregestational and Gestational Diabetes in Saudi Women: Analysis from Riyadh Mother and Baby Cohort Study (RAHMA)

**DOI:** 10.1155/2017/6878263

**Published:** 2017-03-12

**Authors:** Hayfaa Wahabi, Amel Fayed, Samia Esmaeil, Heba Mamdouh, Reham Kotb

**Affiliations:** ^1^Chair for Evidence-Based Health Care and Knowledge Translation, College of Medicine, King Saud University, Riyadh, Saudi Arabia; ^2^Department of Community and Family Medicine, King Saud University, Riyadh, Saudi Arabia; ^3^Department of Biostatistics, High Institute of Public Health, Alexandria University, Alexandria, Egypt; ^4^College of Medicine, Princess Nourah Bint Abdulrahman University, Riyadh, Saudi Arabia; ^5^Department of Family Health, High Institute of Public Health, Alexandria University, Alexandria, Egypt; ^6^Department of Primary Health Care, High Institute of Public Health, Alexandria University, Alexandria, Egypt

## Abstract

The objectives of this study were to estimate the burden of diabetes and to explore the adverse pregnancy outcomes associated with pregestational diabetes mellitus (pre-GDM) and gestational diabetes mellitus (GDM) among the Saudi pregnant population. In this subcohort, we compared the maternal and the neonatal outcomes of diabetic women with pre-GDM and GDM to the outcomes of nondiabetic mothers who delivered during the same period. From the total cohort, 9723 women participated in this study. Of the participants, 24.2% had GDM, 4.3% had pre-GDM, and 6951 were nondiabetic. After adjustment for confounders, women with GDM had increased odds of delivering a macrosomic baby (OR: 1.6; 95% CI: 1.2–2.1). Women with pre-GDM were more likely to deliver by Cesarean section (OR: 1.65; CI: 1.32–2.07) and to have preterm delivery < 37 weeks (OR: 2.1; CI: 1.5–2.8). Neonates of mothers with pre-GDM were at increased risk of being stillbirth (OR: 3.66; CI: 1.98–6.72), at increased risk of admission to NICU (OR: 2.21; CI: 1.5–3.27), and at increased risk for being macrosomic (OR: 2.40; CI: 1.50–3.8). The prevalence of GDM and pre-GDM in the Saudi pregnant population is among the highest in the world. The conditions are associated with high maternal and neonatal morbidities and mortalities.

## 1. Introduction

According to the International Diabetes Federation (IDF), the epidemiology of diabetes during pregnancy is unknown in many countries in the world [1]. Nevertheless, more than 21 million pregnancies were affected by diabetes during the year 2013 [1]. Saudi Arabia is among the top ten countries in the world with the highest prevalence of diabetes [1, 2]. A recent report from Saudi Arabia estimated the prevalence of pregestational diabetes mellitus (Pre-GDM) and gestational diabetes mellitus (GDM) in Riyadh, the capital city of Saudi Arabia, to be 4.3% and 24.3%, respectively [3]. This prevalence reflects high burden of diabetes among pregnant women compared to other populations in the world [4, 5].

Pregnancies complicated by maternal diabetes are associated with adverse maternal and neonatal outcomes including increased rate of Cesarean section delivery, macrosomia, admission to neonatal intensive care unit (NICU), and perinatal mortality [6, 7]. Interventions such as preconception care for women with pre-GDM and screening and control of hyperglycemia during pregnancy for women with GDM have been proven to improve the outcomes for pregnancies complicated with diabetes [8–10]. Hence, it is important to estimate the burden of diabetes and its complication among pregnant women to direct health resource to improve the outcomes for these high-risk pregnancies.

We used data from a subcohort of RAHMA study to estimate the burden of diabetes among pregnant women and to explore the adverse pregnancy outcomes associated with pre-GDM and GDM in Saudi pregnant population.

## 2. Methods

### 2.1. Study Setting

RAHMA is the first large multicenter cohort study which investigates pregnancy outcomes in Saudi Arabia. Because RAHMA has systematically recruited a large number of pregnant women, it is expected to provide accurate estimate of the indices of maternal morbidity in Riyadh and to a great extent in Saudi Arabia generally, bearing in mind that more than 25% of the Kingdom's population (7.3 million) lives in Riyadh [11] and that all women deliver in hospitals. The participating hospitals were selected randomly after stratification based on the type of hospital (ministry of health, military, or teaching) and the number of beds which ensured that the participants in the study were representatives of all the spectra of pregnant women in Riyadh. The detailed methodology of the study has been previously reported [3].

The main objectives of the study were to examine the influence of noncommunicable diseases including diabetes, hypertension, and obesity as well as the effect of the socioeconomic factors such as urbanization, education, and smoking on the outcomes of pregnancy. RAHMA recruited 14,568 women during the period from 2013 to 2015. Participants completed a self-administered questionnaire providing information on family socioeconomic and lifestyle status and antenatal history. In addition, maternal and neonatal outcomes were reported. A link was established between maternal laboratory data and the study records [3].

### 2.2. Study Population

In this subcohort, we compared the maternal and the neonatal outcomes of diabetic women with pre-GDM (type 1 and type 2) and GDM to the outcomes of nondiabetic mothers who delivered during the same period. In addition, we compared the outcomes of pregnancies complicated with type 1 diabetes mellitus (T1DM) to those complicated with type 2 diabetes mellitus (T2DM).

Due to the variable cut-off values for the diagnosis of GDM and pre-GDM in the three participating hospitals, we collected the results of the OGTT between 24 and 34 gestation weeks and the fasting blood glucose ≤ 14 weeks of gestation to reclassify the participants as nondiabetic, pre-GDM, or GDM based on the following World Health Organization (WHO) cut-off values [12]:

  Gestational diabetes mellitus should be diagnosed at any time in pregnancy if one or more of the following criteria are met:
  - Fasting plasma glucose 5.1–6.9 mmol/l (92–125 mg/dl).
  - 1-Hour plasma glucose ≥ 10.0 mmol/l (180 mg/dl) following a 75 g oral glucose load.
  - 2-Hour plasma glucose 8.5–11.0 mmol/l (153–199 mg/dl) following a 75 g oral glucose load.
  Diabetes in pregnancy should be diagnosed if one or more of the following criteria are met:
  - Fasting plasma glucose ≥ 7.0 mmol/l (126 mg/dl).
  - 2-Hour plasma glucose ≥ 11.1 mmol/l (200 mg/dl) following a 75 g oral glucose load.
  - Random plasma glucose ≥ 11.1 mmol/l (200 mg/dl) in the presence of diabetes symptoms.

Maternal prepregnancy body mass index (BMI) was calculated from maternal recall of weight prior to pregnancy and the height measured during the first antenatal clinic and then the participants were classified according to the WHO weight classification. The following BMI definitions were used in this study: underweight (<18.5 kg/m^2^), normal (18.5–24.9 kg/m^2^), overweight (25.0–29.9 kg/m^2^), and obese (≥30 kg/m^2^) [13].

For the purpose of this study, preeclampsia was defined as new onset of elevated blood pressure after 20 weeks of pregnancy in a previously normotensive woman (≥140 mmHg systolic or ≥90 mmHg diastolic on at least two occasions 6 h apart) in addition to proteinuria of at least 1+ on a urine dipstick or ≥300 mg in a 24-hour urine collection. Eclampsia is defined as seizures in a preeclamptic woman that cannot be attributed to other causes. Gestational hypertension is defined as new onset of elevated blood pressure (≥140 mmHg systolic or ≥90 mmHg diastolic on at least two occasions 6 h apart) after 20 weeks of gestation in a previously normotensive woman and superimposed preeclampsia is defined as new onset of preeclampsia after 20 weeks of pregnancy in a previously hypertensive woman [14].

The inclusion criteria for this subcohort were the following:

1. Gestational age of 24 weeks or more at the time of delivery, calculated from the last menstrual period and/or early ultrasound scan
2. Singleton pregnancy
3. Women diagnosed with either T1DM or T2DM before the index pregnancy or according to the WHO criteria during index pregnancy and women with GDM (study groups)
4. Women with neither pre-GDM nor GDM (control group)

We excluded women with unknown glycemic status from the analysis.

The demographic characteristics and the pregnancy outcomes of the women with pre-GDM and GDM were compared to the outcomes of nondiabetic women. Women with multiple pregnancies were excluded from the analysis of the outcomes; however, all deliveries more than 24 weeks were considered when calculating the prevalence rate of pre-GDM and GDM.

### 2.3. Outcomes

The maternal variables we compared were age, parity, induction of labour, mode of delivery, premature delivery, rate of hypertensive disorders in pregnancy, and prepregnancy BMI. The neonatal outcomes included birth weight, macrosomia (birth weight ≥ 4 kg), low birth weight, shoulder dystocia, APGAR scores at 5 min after delivery, admission to neonatal intensive care unit (NICU), and the prevalence of stillbirth.

### 2.4. Statistical Analysis

Data were analyzed using Statistical Package for the Social Sciences (SPSS), Version 20 (SPSS Inc., Chicago, IL, USA). ANOVA test was used to compare means and Chi squire test was used to compare categorical variables between the three groups. Odds Ratio (OR) was calculated and *P* value of less than 0.05 was considered significant. We used regression analysis to adjust for covariates including maternal age, BMI, and parity. Nondiabetic women were taken as reference group.

### 2.5. Ethical Approval

The Review Boards of the following institutions reviewed and approved the main cohort study (RAHMA) from which a subgroup population was included in this study: King Abdullah International Medical Research Centre (approval letter: 11/062), King Fahad Medical City Research Centre (approval letter: 013–017), and King Saud University (approval letter: 13–985). The study was conducted according to the principles expressed in the Declaration of Helsinki.

## 3. Results

From the total cohort, 9723 women had OGTT and were included in this study, while 4845 were not screened giving a screening rate of 66.7%. The reasons for the missing OGTT results were as follows: 2695 participants (18.5%) did not have the test because they either did not book for antenatal care or booked after 34 weeks of gestation, 1530 participants (10.5%) were screened for GDM during their antenatal care in hospitals other than those participating in the study and the results were not available, and the remaining 582 participants (4%) declined to have the test for various reasons.

Comparison of the main demographic characteristics and determinants of GDM between women who had OGTT test results and those who did not showed no systematic difference between the two groups (see Appendix).

**Figure 1:**
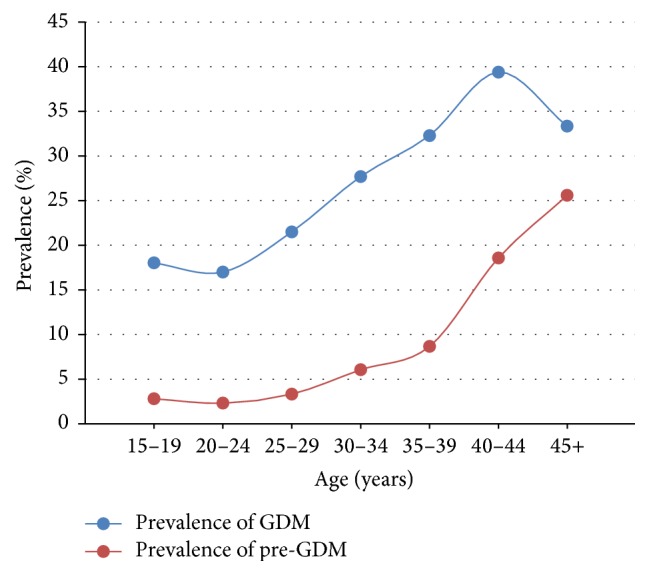
Prevalence of gestational and pregestational diabetes by age range.

Of the participants of the study, 2354 (24.2%) had GDM, 418 (4.3%) had pre-GDM, and 6951 were nondiabetic.  Table 1 shows the comparison between the demographic characteristics of the three groups. There were no significant differences between the three groups in the socioeconomic characteristics. However, women with GDM and pre-GDM were significantly older and of higher parity when compared to nondiabetic women (Table 1). While less than 20% of women between 20 and 24 years were diabetic, we found that more than 45% of women with 45 years or more were diabetic (Table 1). In addition, the prevalence of GDM and pre-GDM increases with the increase of maternal age, with slight drop in the prevalence of GDM after 44 years of age (Figure 1). While more than 75% of women with GDM were between 25 and 39 years, more than 70% of women with pre-GDM were between 30 and 44 years, which indicates a later onset of pre-GDM compared to GDM (Table 1).

Comparison of the maternal and neonatal outcomes between women with pre-GDM, those with GDM, and nondiabetic women is shown in Table 2. The pregnancy outcomes for women with GDM were comparable to the outcomes of nondiabetic women except for macrosomia (Table 2). However, women with pre-GDM had significantly increased proportion of adverse pregnancy outcomes compared to nondiabetic women, including preterm delivery between 34 and 36 weeks, stillbirth rate, neonatal admission to intensive care unit (NICU), macrosomia, low APGAR scores, and shoulder dystocia (Table 2). In addition, higher proportion of women with pre-GDM had hypertensive disorders during pregnancy, induction of labour, and Cesarean section delivery (Table 2).

After adjustment for maternal age, parity, and BMI, women with GDM had increased odds of delivering a macrosomic baby (Odds Ratio (OR): 1.6; 95% confidence interval (CI): 1.2–2.1) compared to nondiabetic women, with no significant increase risk for other maternal or neonatal complications. However, women with pre-GDM continued to have increased risk of developing complication after adjustment. They were more likely to have induction of labour (OR: 1.67; CI: 1.28–2.13), to deliver by Cesarean section (OR: 1.65; CI: 1.32–2.07), and to have preterm delivery < 37 weeks (OR: 2.1; CI: 1.5–2.8) compared to nondiabetic women. Neonates of mothers with pre-GDM were at more than threefold increased risk of being stillbirth (OR: 3.66; CI: 1.98–6.72), at nearly fourfold increased risk of having low APGAR scores at birth (OR: 3.82; CI: 2.26–6.45), at more than twofold increased risk of admission to NICU (OR: 2.21; CI: 1.5–3.27), and at more than twofold increased risk for macrosomia (OR: 2.40; CI: 1.50–3.8), compared to neonates of nondiabetic mothers (Table 3).

**Figure 2:**
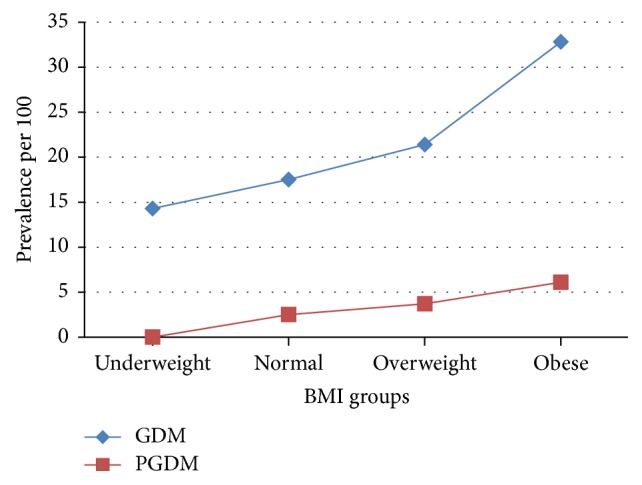
Prevalence of gestational diabetes and pregestational diabetes and body mass index groups.

The relationship between the prevalence of GDM and pre-GDM and BMI is shown in Figure 2. Both conditions showed increased prevalence with increase in BMI.

The comparison of the maternal characteristics and the neonatal outcomes between women with T1DM and T2DM and the women diagnosed as diabetic during the index pregnancy is shown in Table 4. Almost 50% of the women with pre-GDM in this subcohort were diagnosed during pregnancy. The three groups were similar in most of the maternal characteristics and pregnancy outcomes except for preterm delivery where women with T1DM had significantly more preterm deliveries compared to the other two groups (Table 4).

## 4. Discussion

Our results showed that almost 30% of the obstetric population in Riyadh suffers from the adverse effects of either pre-GDM or GDM and that nearly 50% of pregnant women with T2DM were unaware of their condition. In addition, the study demonstrated the increased morbidities and mortalities associated with pre-GDM compared to GDM and between T1DM and T2DM.

The high prevalence of 24% and 4.3% for GDM and pre-GDM, respectively, is quite alarming, considering the high risk for adverse pregnancy outcomes associated with diabetes in pregnancy. Although our estimated prevalence is consistent with that detected by global estimates of GDM and pre-GDM in the region and from similar studies in Saudi Arabia [5–7, 15], it is much higher than the prevalence reported from USA and Europe [5]. Such finding is expected due to the established relationship between ethnicity and epidemiology of pre-GDM and GDM [16, 17].

Moreover, such high rate of GDM and pre-GDM is anticipated in Saudi pregnant women, considering the high burden of diabetes in the adult population in the country [18] and the fact that epidemiology of GDM is directly related to that of T2DM in the population [19].

Some of the recognized risk factors for the development of GDM and pre-GDM are well documented in this study, including increase in maternal age and obesity.

The association between advance age and development of GDM and pre-GDM is well established [20]. Our results showed increase in the prevalence of GDM and pre-GDM as the maternal age advances (Figure 1). This finding is consistent with previous studies from Saudi Arabia and other parts of the world for diabetes in pregnant and in nonpregnant subjects [6, 7, 21]. The slight drop in the prevalence of GDM at maternal age of 45 years is explained by the small number of pregnancies at this category of age and the sharp increase in the prevalence of pre-GDM to reach the national prevalence of 25% [18] (Figure 1).

Obesity is a risk factor for many maternal and perinatal adverse outcomes, including increased Cesarean section delivery, hypertensive disorders in pregnancy, macrosomia, and perinatal mortality [22, 23]. In addition, obesity is associated with the development of T2DM and GDM due to the increased peripheral resistance to insulin [24].

In this study, the burden of overweight and obesity is very high in the whole cohort and especially among diabetic women. While as much as 65% of the nondiabetic women were either obese or overweight, the corresponding proportions of women with diabetes were 78 and 82% for GDM and pre-GDM, respectively. Furthermore, the prevalence of diabetes in the cohort increased with increase in BMI (Figure 2).

However, when we controlled for obesity as a covariant for adverse maternal and neonatal outcomes, pre-GDM continued to be a risk factor for all adverse outcomes except for shoulder dystocia, while the effect of GDM was attenuated to become a risk for no complication other than macrosomia (Table 3).

Few published studies compared the effects of GDM to those of pre-GDM [25, 26]. Our results are consistent with the previous report which demonstrates that pre-GDM confers higher risk for adverse pregnancy outcomes including nearly fourfold increase in the risk of stillbirth and fetal distress and more than twofold increase in the risk of preterm birth and admission to NICU (Table 3). This can be explained by the longer exposure of the fetus to more severe hyperglycemic environment than in the case of GDM. The linear relationship between the degree of hyperglycaemia and the development of certain maternal and neonatal complications was recently established by the HAPO study and similar reports [27, 28].

Although the frequency of structural congenital abnormalities detected by mid-trimester ultrasound scan was more in the pre-GDM group compared to the other groups (Table 2), we expected to find higher rate. That expectation is based on the known teratogenic effect of pre-GDM and the fact that nearly 50% of the women in this cohort were diagnosed as T2DM at the time of screening and had received no treatment early in pregnancy [29]. Nevertheless, this estimate is associated with uncertainties such as the unknown sensitivity of the mid-trimester ultrasound scan in any center in Saudi Arabia for the detection of structural abnormalities.

Unfortunately, it will be difficult to link congenital anomalies detected in the postnatal period to any maternal condition due to lack of national registry for congenital anomalies and the difficulty of following a cohort of children with the frequent change of address and healthcare provider.

Similar to most of the published studies, we found no differences in terms of the complications we investigated between women with T1DM and T2DM except for preterm delivery (Table 4) [30, 31]. Contrary to our expectations, we found similar frequency of adverse outcome reported for women known to have T2DM and those diagnosed during the index pregnancy. This may be explained by the shorter duration of the T2DM prior to the index pregnancy for women who did not know they had the condition and the lack of preconception control for women known to have the condition.

## 5. Implication to Practice

Although our estimated risks for maternal, fetal, and neonatal complications of pre-GDM and GDM are similar to other studies [21, 23, 25, 26, 32], the impact on the Saudi pregnant population is expected to be bigger due to the high burden of these conditions in the community, which calls for immediate, evidence-based measures including the following:

1. Preconception care should be integrated in the healthcare of Saudi diabetic women considering the proven effects associated with such services in reducing all the complications of pre-GDM [9].
2. Early screening during pregnancy, preferably early in the first trimester, for pre-GDM, is a justified strategy of care, especially when 50% of women with T2DM in this study were diagnosed during pregnancy.
3. Establishing national program for screening adults, including women in reproductive age, for diabetes and prediabetic state is a prudent step towards controlling such an epidemic. To reduce the cost of such a program, selective screening policy based on at-risk population, for example, high BMI, should be adopted.
4. As diabetes in pregnancy is a major public health problem, it is mandatory to establish national guidelines for the screening and management of GDM and pre-GDM, which will standardize care, improve outcomes [8, 9], and provide the opportunity for monitoring.
5. Establishment of national registries for main adverse effects of diabetes in pregnancy such as for congenital abnormalities, perinatal mortality, and Caesarean section will facilitate monitoring of interventions and will enhance audit and research.
6. Postpartum screening of women with history of GDM for hyperglycemia and T2DM provides an opportunity for reducing the prevalence of T2DM and its complications.
7. Effective strategies should be implemented to reduce weight gain and obesity during pregnancy and in the postpartum period [33, 34].
8. Saudi women in the reproductive age group should receive health education about the serious adverse effects of diabetes and obesity on their reproductive life. Such health education can be integrated in school and university education as over 90% of the women are attending schools or universities.

## 6. Implication to Research

Further research should be directed to the investigation of the prevalence of T2DM and prediabetic state following gestational diabetes and to the effects of maternal hyperglycaemia during pregnancy on the infant and the future adult health considering the proven ill effects of these conditions on the future adult [35, 36]. In addition, research should be directed towards effective interventions to reduce the burden of obesity during pregnancy and its adverse effects.

## 7. Strength and Limitations of the Study

Our study is the first prospective report on a large cohort of nearly 10,000 participants, recruited from three centers, for the investigation of GDM and pre-GDM in the Middle East. An important strength of the study is the inclusion of a nondiabetic group in the analysis and the comparison between T1DM and T2DM. In addition, we have minimized the possibility of misclassification through reclassification of participants into GDM, pre-GDM, or nondiabetic groups, according to the WHO guidelines of diabetes in pregnancy, irrespective of the ICD coding, which increased the reliability of our results. The study investigated the effect of diabetes on many important maternal and neonatal outcomes and considered known covariates in the analysis to estimate the true effect of diabetes on the mother and the infant.

We acknowledge the limitations of this study including the fact that more than 4800 women were excluded from the study because they were not screened for diabetes. In addition, the BMI was calculated from the self-reported prepregnancy weight, which might have underestimated the prevalence of obesity and overweight. However, no significant difference in the prevalence of obesity was found when interpregnancy weight was estimated in the original cohort [3].

## 8. Conclusion

The prevalence of GDM and pre-GDM in the Saudi pregnant population is among the highest in the world. The conditions are associated with high maternal morbidities and fetal and neonatal morbidities and mortalities. Immediate effective interventions are needed to reduce the burden of diabetes in pregnancy.

## Figures and Tables

**Table 1:** The demographic characteristics of women with pregestational diabetes, those with gestational diabetes, and nondiabetic women.

Characteristic	Nondiabetic Number (%) *N* = 6951 (71.5)	GDM Number (%) *N* = 2354 (24.2)	Pre-GDM Number (%) *N* = 418 (4.3)	*P* value
Age (mean ± SD)	29.5 ± 5.7	31.5 ± 5.9	33.6 ± 6.2	<0.001
Age range (years)				
<20	173 (2.5)	38 (1.6)	5 (1.2)	
20–24	1322 (19)	271 (11.5)	32 (7.7)	
25–29	2179 (31.3)	596 (25.3)	75 (17.9)	
30–34	1799 (25.9)	688 (29.2)	116 (27.8)	
35–39	1126 (16.2)	537 (22.8)	106 (25.4)	
40–44	320 (4.6)	208 (8.8)	73 (17.5)	
45+	32 (0.5)	16 (0.7)	11 (2.6)	
Education				
Illiterate	123 (3.0)	40 (3.0)	5 (2.1)	0.26
School	2259 (54.7)	727 (55.3)	145 (62.2)	
University and above	1747 (42.3)	547 (41.6)	83 (35.6)	
Missing	2822	1040	185	
Working status				
Housewife	5179 (86.5)	1754 (86.7)	317 (88.8)	0.39
Employed	765 (12.8)	251 (12.4)	40 (11.2)	
Student	46 (0.8)	18 (0.9)	0 (0.0)	
Missing	961	331	61	
Parity				
Nullipara	1655 (23.8)	378 (16.1)	64 (15.3)	<0.01
2–4	3351 (48.2)	1090 (46.3)	130 (31.1)	
Grand multipara	1941 (27.9)	885 (37.6)	224 (53.6)	
Missing	4	1	0	
BMI before pregnancy (kg/m^2^)				
Less than 18.5	96 (2.6)	16 (1.3)	0 (0.0)	<0.01
18.5 to 25	1200 (32.4)	263 (20.8)	38 (17.5)	
25.1 to <30	1227 (33.1)	351 (27.8)	61 (28.1)	
30 or more	1179 (31.8)	633 (50.1)	118 (54.4)	

Data expressed as mean ± SD or *N* (%). BMI: body mass index; GDM: gestational diabetes mellitus; Pre-GDM: preexisting gestational diabetes mellitus.

**Table 2:** Comparison of the maternal and neonatal outcomes between women with pregestational diabetes, those with gestational diabetes, and nondiabetic women.

Characteristic	Nondiabetic *N* = 6951 (71.5)	GDM *N* = 2354 (24.2)	Pre-GDM *N* = 418 (4.3)	*P* value
Pregnancy				
Single	6763 (97.3)	2282 (96.9)	403 (96.4)	0.42
Multiple	188 (2.7)	72 (3.1)	15 (3.6)	
Gestational age (for live births)				
24–34 weeks	143 (2.1)	59 (2.5)	11 (2.7)	<0.01
34–36 weeks	398 (5.8)	137 (5.9)	48 (11.9)	
37–41 weeks	6190 (90.6)	2092 (90.3)	341 (84.4)	
More than 41 weeks	104 (1.5)	30 (1.3)	4 (1.0)	
Induced labour	1108 (16.0)	420 (17.9)	95 (22.8)	<0.001
Birth weight (full term)				
Normal (2.5–3.9 kg)	5770 (91.6)	1906 (89.6)	311 (88.4)	<0.001
LBW < 2.5 kg	374 (5.9)	118 (5.5)	19 (5.4)	
Macrosomia ≥ 4.0 kg	156 (2.5)	103 (4.8)	22 (6.2)	
Stillbirth	60 (0.9)	22 (0.9)	12 (2.9)	<0.01
Shoulder dystocia	26 (0.4)	8 (0.3)	5 (1.2)	0.03
Neonatal admission to NICU	281(4.1)	110 (4.7)	33 (8.0)	0.001
APGAR < 7 (for full term)	42 (0.7)	21 (1.0)	11 (3.1)	<0.001
Hypertension disorder				
Preexisting hypertension	69 (1.0)	32 (1.4)	18 (4.3)	<0.01
Gestational hypertension	90 (1.3)	43 (1.8)	18 (4.3)	<0.01
Preeclampsia/superimposed	74 (1.1)	24 (1.0)	9 (2.2)	0.12
Mode of delivery				
Vaginal	4958 (71.9)	1585 (67.6)	239 (57.5)	<0.01
Instrumental	284 (4.1)	84 (3.6)	11 (2.6)	
Cesarean section	1657 (24.0)	675 (28.8)	166 (39.9)	
Structural anomalies detected by antenatal USS	97 (1.4)	35 (1.5)	8 (1.9)	0.67

Data expressed as *N* (%). GDM: gestational diabetes mellitus; Pre-GDM: preexisting gestational diabetes mellitus; NICU: neonatal intensive care unit; LBW: low birth weight; USS: ultrasound scan.

**Table 3:** Crude and adjusted Odds Ratio for maternal and neonatal complications in diabetic women compared to nondiabetic women.

Outcome	GDM *N* = 2354 (24.2)		Pre-GDM *N* = 418 (4.3)	
	Crude OR (95% CI)	Adjusted OR (95% CI)	Crude OR (95% CI)	Adjusted OR (95% CI)
Cesarean section	1.28 (1.1–1.4)^*∗*^	1.05 (0.94–1.18)	2.1 (1.7–2.5)^*∗*^	1.65 (1.32–2.1)^*∗*^
Induction of labour	1.1 (1.01–1.3)^*∗*^	1.39 (1.0–1.3)	1.55 (1.2–1.9)^*∗*^	1.67 (1.28–2.1)^*∗*^
Preterm delivery < 37 weeks	1.1 (0.9–1.3)	1.0 (0.8–1.3)	2.0 (1.5–2.7)^*∗*^	2.1 (1.5–2.8)^*∗*^
Stillbirth	1.08 (0.66–1.77)	1.1 (0.68–1.78)	3.39 (1.8–6.3)^*∗*^	3.66 (1.9–6.7)^*∗*^
Macrosomia	2.1 (1.6–2.7)^*∗*^	1.6 (1.2–2.1)^*∗*^	2.7 (1.7–4.4)^*∗*^	1.6 (1.1–2.7)^*∗*^
Shoulder dystocia	0.9 (0.4–2.0)	1.0 (0.41–2.43)	3.2 (1.2–8.4)^*∗*^	2.4 (0.7–8.5)
APGAR < 7 at 5 min	1.4 (0.87–2.5)	1.25 (0.84–1.86)	4.4 (2.2–8.7)^*∗*^	3.82 (2.26–6.45)^*∗*^
Admission to NICU	1.2 (0.96–1.51)	1.16 (0.91–1.48)	2.12 (1.5–3.1)^*∗*^	2.21 (1.5–3.27)^*∗*^

OR: Odds Ratio; GDM: gestational diabetes mellitus; Pre-GDM: preexisting gestational diabetes mellitus; NICU: neonatal intensive care unit.

OR is adjusted for maternal age, parity, and BMI. Nondiabetic women are reference group.

^*∗*^ *P* value < 0.05.

**Table 4:** Comparison of the maternal characteristics and neonatal outcomes between women with type 1 and type 2 diabetes mellitus.

Characteristic	Type 1 (*n* = 73)	Type 2 (*n* = 141)	Diagnosed during pregnancy (*n* = 204)	*P* value
Age	33.1 ± 6.3	35.3 ± 5.7	34.8 ± 5.0	0.02
BMI	34.5 ± 6.0	35.9 ± 7.4	36.1 ± 6.5	0.22
Parity	3.1 ± 2.7	4.1 ± 2.7	3.5 ± 2.6	0.02
Cesarean section delivery	34 (46.6)	79 (56.8)	98 (48.8)	0.24
Preterm delivery				
24–33 weeks	4 (5.6)	5 (3.5)	7 (3.4)	0.74
34–36 weeks	19 (26.4)	25 (17.7)	25 (12.3)	0.02
Stillbirth	3 (4.1)	4 (2.8)	7 (3.4)	0.88
Macrosomia	4 (5.6)	12 (8.6)	4 (2.0)	0.08
APGAR < 7 at 5 minutes	1 (1.4)	6 (4.3)	9 (4.5)	0.49
Shoulder dystocia	0 (0.0)	1 (0.7)	4 (2.0)	0.59
Admission to NICU	8 (11.0)	15 (10.8)	13 (6.5)	0.29

Data expressed as mean ± SD or *N* (%). BMI: body mass index; NICU: neonatal intensive care unit.

**Table 5:** Comparison of the main demographic characteristics and determinants of GDM between women who had OGTT test results and those who did not.

Characteristic	Women with data available for glycemic classification Mean ± SD	Women with missing data for glycemic classification Mean ± SD
Mother's Age (years)	30.2 ± 5.9	29.3 ± 5.9
Number of pregnancies	3.7 ± 2.5	3.4 ± 2.4
Number of deliveries	2.3 ± 2.1	2.2 ± 2.1
Mother's weight on delivery (kg)	78.7 ± 14.8	77.9 ± 14.6
Gestational age at delivery (weeks)	38.6 ± 2.2	38.4 ± 2.5
Pregnancy BMI (kg/m^2^)	31.8 ± 5.8	31.4 ± 5.6
Prepregnancy BMI (kg/m^2^)	28.4 ± 5.6	27.8 ± 5.5

## Appendix

See Table 5.
